# Parents, time, and space: The three dimensions of endosperm gene expression

**DOI:** 10.1093/plphys/kiac566

**Published:** 2022-12-09

**Authors:** Sergio Galindo-Trigo

**Affiliations:** Section for Genetics and Evolutionary Biology, Department of Biosciences, University of Oslo, 0316 Oslo, Norway

The evolutionary innovation of the seed fueled the expansion of flowering plants by providing protection and expanding the dispersal range of their offspring (Baroux and Grossniklaus, 2019). Angiosperm seeds encapsulate the products of double fertilization: the embryo and endosperm. While the embryo develops into the new plant, the endosperm contributes to the embryo's development and the seedling's success post-germination.

Seeds accumulate large amounts of nutrients, and the endosperm is a key player in this process. In staple cereal grains like maize (*Zea mays*), rice (*Oryza sativa*), or wheat (*Triticum aestivum*), the endosperm persists through seed maturation to provide nourishment for the germinating seedling. In these species, the endosperm constitutes the largest seed storage compartment, highlighting its importance for human and livestock nutrition (Liu et al., 2022). In the case of most dicotyledonous plants, however, the endosperm degrades during seed maturation and its nutrients instead accumulate in the cotyledons or embryonic leaves.

**Figure 1:**
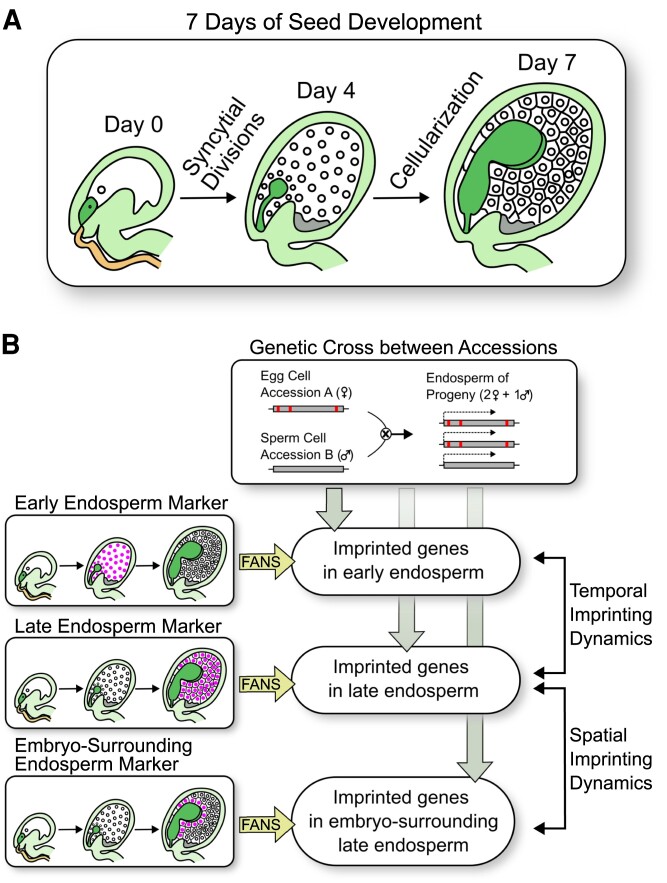
Early stages of seed development and methodology to investigate spatiotemporal genomic imprinting in the endosperm in Arabidopsis. A, Simplified representation of the first 7 days of endosperm development (in orange, pollen tube; in dark green, zygote/embryo; in white, endosperm; in gray, chalazal endosperm). B, On the left, diagrams representing each of the three reporter lines with fluorescently tagged nuclei (early endosperm, total late endosperm, and embryo-surrounding late endosperm). At the top of the central column, the genetic cross between two Arabidopsis accessions is represented with any given gene as a gray box, showing polymorphisms between accessions in red, and the resulting triploid endosperm genotype with two maternal and one paternal copies of the genome; Below, the combination of the reporter lines, FANS, and bioinformatic analysis of the endosperm transcriptome resulting from the accessions' cross allows the identification of imprinted genes in the originating fluorescently-tagged nuclei. On the right, comparison of the imprinted genes at different timepoints and different endosperm domains yields the spatial and temporal analysis of endosperm imprinting dynamics. Figure created by Sergio Galindo-Trigo based on van Ekelenburg et al. (2021).

The endosperm undergoes a distinct developmental program as the seed matures and the embryo develops. In some dicotyledonous plants like the model organism Arabidopsis (*Arabidopsis thaliana*), the endosperm first grows through nuclear multiplication without cytokinesis into a syncytium (which is by definition a multinucleated cell), after which the endosperm cellularizes. At this point, the endosperm starts to behave as a nutrient source for the developing embryo (Figure 1A). Finally, the endosperm progressively degrades as the embryo expands inside the seed (Li and Berger, 2012).

Different regions of the endosperm acquire distinct functional identities as evidenced by discreet subdomains of gene expression within the endosperm. Functional specialization of endosperm domains implies that this genetically uniform tissue communicates with its neighboring compartments (i.e. embryo, seed coat) and responds to additional cues such as nutrient gradients and mechanical constraints to ensure seed development is appropriately orchestrated (Yan et al., 2014).

As a product of double fertilization, the endosperm carries genetic information from both progenitors and is thus under their direct genetic influence. In most flowering plants, the endosperm is triploid and carries three copies of the genome, two maternal and one paternal, meaning that its development is also impacted by the presence and dosage of specific parental alleles. Genomic imprinting, the preferential expression of a specific parental allele of any given gene, is an additional mechanism controlling endosperm gene expression. Imprinting is executed at the male and female gametes through epigenetic modifications (DNA methylation, histone tail modifications, etc.) that persist after fertilization. In plants, the endosperm is the tissue in which imprinting preferentially takes place and alterations in the epigenetic machinery that regulates imprinting result in aberrant endosperm and seed development (Gehring and Satyaki, 2017). Understanding how imprinting mechanistically affects endosperm development is thereby necessary for genome editing programs targeted at improving seed quality.

In the current issue of *Plant Physiology*, van Ekelenburg et al. report on the dynamic imprinting of the Arabidopsis endosperm, providing temporal and spatial resolution of parent-of-origin allelic expression (van Ekelenburg et al., 2022). Studying the endosperm is methodologically challenging as it is deeply embedded in maternal tissues with distinct genomic identities, and genetic investigation is often hindered by lethality of knock-out mutations. Indeed, previous efforts to understand endosperm imprinting revealed contamination from surrounding maternal tissues (Schon and Nodine, 2017).

To avoid this, van Ekelenburg et al. used the fluorescence-activated nuclear sorting (FANS) method, obtaining endosperm domain-enriched samples for transcriptomic analyses. Gene promoters displaying specific temporal and spatial endosperm expression patterns were identified and transformed into the Arabidopsis accession Col-0 to produce reporter lines that fluorescently tag nuclei during early (precellularization) total endosperm development, late (postcellularization) total endosperm, and late embryo-surrounding endosperm. The authors generated hybrid seeds between the Col-0 endosperm reporter lines and a second Arabidopsis accession, Tsu-1. By using different Arabidopsis accessions as male or female parents, the authors were able to identify the parental origin of each allele in the transcriptomic dataset (based on their known polymorphisms). The use of FANS to enrich samples in specific endosperm domains from the respective reporter lines at two timepoints added spatial and temporal components to the analysis (Figure 1B). This multidimensional transcriptomic dataset demonstrated that endosperm imprinting is spatiotemporally regulated, therefore introducing an additional layer of complexity to this phenomenon.

Furthermore, several genes displayed an intriguing imprinting pattern: they were biparentally expressed at early stages but imprinted later. These results suggest a mechanism that primes specific alleles and spreads through multiple rounds of genome duplication and nuclear division until the imprinting mark manifests. Such temporal imprinting profile challenges the canonical imprinting view under which the epigenetic changes are already established in the gametes prefertilization.

The research presented by van Ekelenburg and colleagues successfully improved on the methodological state-of-the-art of endosperm imprinting studies and provided the community with evidence for spatial and temporal imprinting. Functionally characterizing the role of the identified spatiotemporally imprinted genes in seed development will be key to mechanistically understand imprinting. As highlighted by the authors, the low rate of overlap of identified imprinted genes between studies with a similar aim highlights the importance of continued implementation of novel methodologies and conceptual replication of scientific studies (Nosek and Errington, 2017). It will therefore be exciting to see how future developments in next-generation sequencing and single-cell sequencing will continue to build on these findings and facilitate harnessing the mechanisms behind endosperm imprinting toward more precise genetic seed improvements in our crops.
